# Organophosphate Flame Retardants in Indoor Dust in the Tampa Bay (Florida) Area

**DOI:** 10.3390/toxics13060508

**Published:** 2025-06-16

**Authors:** Adebayo Solanke, Lukasz Talalaj, Claire Graham, Henry Alegria

**Affiliations:** 1 Department of Environmental Science, Geography & Policy, University of South Florida St. Petersburg, 140 7th Avenue South, St. Petersburg, FL 33701, USA; adebayo.solanke@fdacs.gov (A.S.); clairegraham514@gmail.com (C.G.); 2 School of the Environment, Florida A&M University, 1601 Martin L. King Jr. Blvd., Tallahassee, FL 32307, USA; 3 Environmental Protection Commission of Hillsborough County, 3629 Queen Palm Drive, Tampa, FL 33619, USA; ltalalaj@usf.edu

**Keywords:** organophosphate flame retardants, indoor dust, exposure, Florida

## Abstract

As polybrominated diphenyl ethers were phased out as flame retardants and plasticizers, increasing quantities of organophosphate triesters (OPEs) have been used as replacements. Despite a surge in reports on levels and profiles of OPEs, especially in indoor environments, and the potential exposure, there are still understudied areas with no data on the levels of these chemicals. We carried out the first study investigating levels and profiles of OPEs in indoor dust from such an area, the Tampa Bay (Florida) area. ∑_13_OPEs measured at each site ranged from 545 to 502,086 ng g^−1^, with overall medians and means over 64 sites of 15,447 and 36,135 ng g^−1^, respectively. Alkyl OPEs were predominant, with lesser levels of chlorinated and aryl OPEs. Median levels were highest for tris (2-butoxyethyl) phosphate (TBOEP) and triphenyl phosphate (TPHP) at 4641 and 1046 ng g^−1^, respectively; lower for tris(1,3–dichloro-2-propyl) phosphate (TDCIPP), tris(2-chloropropyl) phosphate (T2CPP), and tris (2-chloroisopropyl) phosphate (TCIPP) at 530, 458, and 360 ng g^−1^, respectively; with others ranging from 2 to 85 ng g^−1^. There were differences in levels in different microenvironments (urban versus suburban; non-residential versus residential; apartments versus single-family homes; daycares versus residences and university rooms; building age; and rooms with different floor material). Estimated daily intakes for median and higher exposure scenarios for ∑_13_OPEs (in ng kg^−1^ bw day^−1^) were 12 and 552 for toddlers and 6 and 451 for adults, respectively. TBOEP accounted for 30% of total intake for toddlers and adults in a mean exposure scenario but 90% for high exposure scenario.

## 1. Introduction

Organophosphate triesters (OPEs) are a class of chemicals that are used extensively as flame retardants, plasticizers, and stabilizers in many consumer products and housing materials and as antifoaming agents in industrial products [1,2,3,4,5,6,7,8,9]. Because of the phase-out of polybrominated biphenyl ethers (PBDEs), which served the same purposes as OPEs, the global use of OPEs increased. Ma et al. (2024) reported that between 2010 and 2020 the production of organophosphate flame retardants increased from 364,300 tons to 504,300 tons and consumption increased from 374,700 tons to 472,900 tons [10]. Du et al. (2024) reported that, in 2022, global consumption of organophosphate flame retardants was approximately 756,000 tons [11].

With the increased use of OPEs came an increased interest in understanding their cycling and fate in the environment, particularly in light of studies suggesting the toxicological effects of some OPEs [12,13]. Because OPEs are used as additives rather than being bound to the materials to which they are applied, they are susceptible to volatilization, wear, and leaching and thus release into the environment [14,15]. Due to the many indoor sources of OPEs, these chemicals are more abundant in indoor environments [4]. Consequently, there is much interest in understanding the levels of and potential exposure routes to OPEs for people in indoor environments [4,15]. Most OPEs are classified as semi-volatile organic compounds that can sorb to particles [4]. Thus, indoor dust has been shown to be an important matrix for human exposure to these chemicals via the ingestion of house dust (including dust settled onto food), inhalation of airborne particles, and dermal adsorption [4,5,6,7,8]. Several reports have documented the presence of OPEs in human urine, blood serum, hair, and nails, indicating chronic exposure to these chemicals [16,17,18,19,20,21,22]. Meeker et al. (2013) reported a significant correlation between OPEs in indoor dust and OPEs in urine, indicating that exposure to OPEs via dust is a delivery mechanism for these chemicals in humans [22].

Despite the number of reports on OPEs, there are still understudied areas where there is no information regarding concentrations in indoor dust and exposure data. Such is the case in Florida. It is important for a truly global understanding of the fate and cycling of OPEs, and potential exposure to these chemicals, that data from any understudied area be generated. The aim of this study was to estimate human exposure to OPEs via dust ingestion in indoor buildings in the Tampa Bay (Florida) area by carrying out a comprehensive assessment of levels of OPEs in indoor dust in different environments (urban and suburban; daycares, residences, universities; different floor coverings in public buildings and residences) and calculating exposure rates.

## 2. Materials and Methods

### 2.1. Sampling

**Figure 1:**
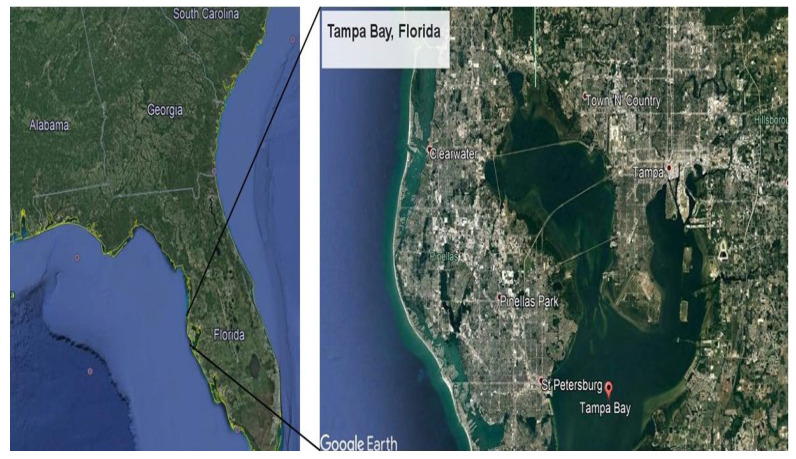
Study area (Tampa Bay, Florida, USA).

A total of 64 indoor dust samples were collected in the Tampa Bay (Florida) area in 2016 from 13 November to 23 December (Figure 1). Samples were collected from different university classrooms and offices, residences (single-family and apartments), and daycares (Table S1).

Dust samples were collected using a hand-held Royal Dirt Devil vacuum cleaner (model 103, Royal Appliance, Cleveland, OH, USA) fitted with individual detachable collection paper bags. At all sampling sites, samples were taken from the floor by vacuuming for 15 min to ensure the collection of sufficient dust for analysis (1.5–2.0 g approximately). Different bags were used for each sample site. In homes, samples were collected from living rooms and/or bedrooms depending upon volunteers’ permission. After vacuuming, each sample bag was detached, sealed, wrapped with clean aluminum foil (baked at 450 °C), placed in a Ziploc bag, and transported to the analytical lab on ice. In the lab, the collected dust was immediately transferred to pre-cleaned amber glass vials with Teflon caps, and the vials were wrapped in aluminum foil and stored in a freezer until analysis.

### 2.2. Chemicals and Reagents

Standards of tris(2-chloroethyl) phosphate (TCEP), tris(2-chloroisopropyl) phosphate (TCIPP), tris(2-chloropropyl) phosphate (T2CPP), tris(1,3–dichloro-2-propyl) phosphate (TDCIPP), triisopropyl phosphate (TIPP), tripropyl phosphate (TPP), tri n-butyl phosphate (TNBP), tris (2-butoxyethyl) phosphate (TBOEP), tris(2-ethylhexyl) phosphate (TEHP), triphenyl phosphate (TPHP), 2-ethylhexyldiphenyl phosphate (EHDPP), o-tricresyl phosphate (ToCP), m-tricresyl phosphate (TmCP), p-tricresyl phosphate (TpCP), and tris(2-isopropyl phenyl) phosphate (TIPPP) were purchased from AccuStandard (CT, USA). Acronyms used follow the recommendations of [23] and for those not therein the acronyms used by [24] are used. Internal standard (^13^C-PCB-105) was purchased from Cambridge Isotope Laboratories (Tewksbury, MA, USA). Isotopically labeled surrogate standards were purchased from Wellington Laboratories (Guelph, ON, Canada). All organic solvents used during analysis were pesticide grade and purchased from Fisher Scientific (Hampton, NH, USA).

### 2.3. Extraction and Analysis

Extraction of OPEs was performed using the method previously described by Ionas and Covaci (2013) [25]. Dust samples were sieved using a 0.15 mm stainless steel mesh sieve. Extraneous material was then removed using a stainless steel tweezer. A subsample of 100 mg of each dust sample was weighed in a glass test tube and spiked with three labeled OPEs (*d*_15_-triethyl phosphate, *d*_27_-tributyl phosphate, and *d*_21_-tripropyl phosphate), vortexed, and left covered for a few hours to complete absorption of the labeled OPEs. The spiked dust was then covered with 5 mL 3:1 hexane/acetone and ultrasonicated for 15 min, followed by ultracentrifugation for 2 min. This procedure was repeated three more times for each sample. The combined extracts were evaporated to near dryness using a gentle stream of ultrapure nitrogen gas and then solvent-exchanged with hexane to a volume of 1 mL. Extracts were cleaned and fractionated by chromatography using Florisil SPE tubes with Teflon 500 mg/3 mL cartridges (Fisher Scientific, Pittsburgh, PA, USA). Cartridges were pre-cleaned and conditioned with 6 mL ethyl acetate followed by 6 mL hexane. The extracts were applied to the cartridges and eluted with 8 mL ethyl acetate. The fraction was evaporated to near dryness using a gentle stream of ultrapure nitrogen and solvent-exchanged into isooctane at a final volume of 500 µL and spiked with 10 μL of the internal standard (^13^C_12_-PCB 105).

Samples were analyzed using triple quadrupole gas chromatography–mass spectrometry operated in electronic impact (GC-MS-EI) mode using an Agilent 7000C GC—7890B MS detector (Agilent Technologies, Santa Clara, CA, USA). Separation was performed using an HP-5MS column (30 m × 250 µm i.d. × 0.25 µm, Agilent Technologies, Santa Clara, CA, USA), with helium carrier gas at a flow rate of 2.25 mL/min. Then, 2 µL of sample volumes was injected splitless. Inlet and transfer line temperatures were 200 °C and 250 °C. The GC oven temperature program was 90 °C for 1 min, ramped to 160 °C at 2 °C/min and 310 °C at 20 °C/min, with a 5 min hold. The ion source temperature was 230 °C.

### 2.4. Quality Assurance and Control

Established laboratory QA/QC procedures were followed to ensure the validity of this study. All sample extractions and processing were performed inside a fume hood without lights. All glassware was baked at 450 °C and solvent-rinsed prior to use. Percent recoveries of the labeled surrogates averaged 82 ± 12%, 76 ± 8%, and 86 ± 10% for *d*_15_-triethyl phosphate, *d*_27_-tributyl phosphate, and *d*_21_-tripropyl phosphate, respectively. Samples were not recovery-corrected based on the labeled surrogates. The method detection limit (MDL) was calculated as the mean of blanks plus 3 standard deviations when OPEs were detected in the dust blanks. MDL values were expressed as concentrations by dividing by 0.1 g. For compounds not detected in blanks, 2/3 of the instrumental detection limits (IDLs) were used for calculating the MDL. IDLs were calculated from the instrument response for the lowest standard to obtain 3:1 signal to noise ratio. MDLs of individual OPEs ranged from 0.2 to 1.2 ng g^−1^. For field blanks *(n* = 8), pre-cleaned anhydrous sodium sulfate was taken to some sampling locations, spread on aluminum foil, vacuumed as a normal sample, and treated by the same method as the samples. TIPP, TPP, TCEP, TBOEP, and T2CPP were detected in blanks at <0.5–1.5% of sample values. For these OPEs, concentrations in samples were adjusted based on these levels. Solvent blanks were run to ensure no contamination via solvents.

## 3. Results and Discussion

### 3.1. Overall OPEs Levels

Fifteen different OPEs were measured in this study. The OPE concentrations detected in dust samples are summarized in Table 1. Table S2 provides details of concentrations at each site. Frequency of detection ranged from 84 to 100% for all OPEs except TIPPP (36%), ToCP (19%), TmCP (25%), and TpCP (30%). The overall (across all 64 sites) median, mean, and range (min–max) of ∑_13_OPEs were 15,447 ng g^−1^, 36,137 ng g^−1^, and (545–502,086) ng g^−1^.

OPEs can be divided into halogenated and non-halogenated, and the latter group can be divided into alkyl versus aryl OPEs. Non-halogenated OPEs were dominant in the Tampa Bay area. For all sites combined, the median, mean, and range (min–max) of non-halogenated OPEs versus halogenated OPEs were 11,049 vs. 2012 ng g^−1^, 32,031 vs. 4104 ng g^−1^, and (426–495,536) vs. (119–46,712) ng g^−1^, respectively. Differences in mean concentrations between non-halogenated versus halogenated OPEs were significantly different (*p* < 0.05, two-sample *t*-test assuming unequal variances). Within the non-halogenated group, alkyl OPEs dominated compared with aryl OPEs: medians of 5453 vs. 1512 ng g^−1^, means of 23,145 vs. 8887 ng g^−1^, and ranges of (101–251,097) vs. (137–244,439) ng g^−1^, respectively. Differences in mean concentrations between alkyl OPEs and aryl OPEs were statistically significant (*p* < 0.05; two-sample *t*-test assuming unequal variances). In terms of relative contribution, halogenated OPEs accounted for approximately 23% of ∑_13_OPEs, while non-halogenated OPEs accounted for almost 77% (55% alkyl OPEs and 22% aryl OPEs).

TBOEP was the most abundant OPE overall, with median (4641 ng g^−1^) and mean (22,672 ng g^−1^) levels that were approximately 4–12 and 3–24 times higher, respectively, than for TPHP, TDCIPP, T2CPP, and TCIPP, the next most abundant OPEs (and up to 3–4 orders of magnitude higher than other OPEs). On average, TBOEP accounted for 51% of ∑_13_OPEs. TBOEP is widely used as a plasticizer in plastics such as PVCs and rubber, a leveler in floor polishes, and a flame retardant in many products. Its high levels are probably explained by such widespread use. TPHP is used as a flame retardant mainly in plasticizers and lubricants in building materials, PVC and LCD screens, and floor waxes. It is usually used as an additive in combination with other flame-retardant mixtures such as Firemaster 550 [7,14,26]. Chlorinated OPEs are applied to rigid and flexible polyurethane foam, so the levels measured may reflect their emission from a variety of consumer products and insulation containing this material [4,25,26]. A Spearman rank-order correlation test showed strong positive correlation between T2CPP/TCIPP (0.99), TpCP/TPHP (0.98), TpCP/TBOEP (0.64), TBOEP/TPHP (0.60), and TmCP/TDCIPP (0.59) and moderate positive correlation between TEHP/EHDPP (0.36), TmCP/TPHP (0.38), TPP/TCIPP (0.32), and TPP/T2CPP (0.32). This suggests common sources for some OPEs across all sampling sites.

These results are similar to results from other regions of the United States and internationally. For example, TBOEP has also been reported as the dominant OPE in house dust in the United States and in other countries [1,2,6,7,27,28,29,30,31,32,33,34,35,36,37]. The relatively high abundance of TPHP, TDCIPP, T2CPP, and TCIPP is also in agreement with previous reports for homes in the U.S. and other countries [1,4,27,30,36,38,39,40]. This suggests that the sources of OPEs in the Tampa Bay region are similar to those in other parts of the country and the world.

### 3.2. Comparison of Urban and Suburban Σιτεσ

**Figure 2:**
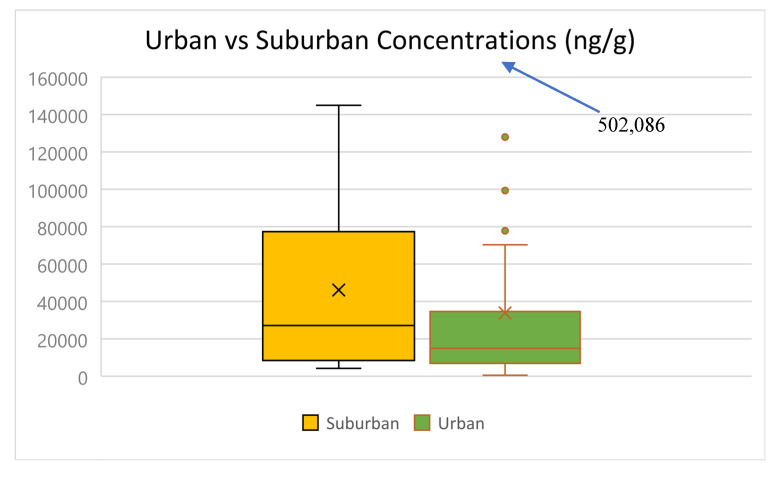
Comparison of ∑_13_OPEs concentrations in urban vs. suburban samples.

Twelve samples were collected from suburban locations and fifty-two were collected from urban locations (using the United States Census Bureau’s definitions of urban and suburban areas). Figure 2 compares the ∑_13_OPEs levels between urban and suburban samples. The overall median, mean, and range (min-max) of ∑_13_OPEs for the urban versus suburban samples were 14,932 versus 27,161 ng g^−1^, 33,840 versus 46,077 ng g^−1^, and (545–502,086) versus (4181–144,966) ng g^−1^, respectively (Table S3). The differences in concentrations were not statistically significant (*p* > 0.05; two-sample *t*-test assuming unequal variances). The higher overall levels of ∑_13_OPEs at suburban locations were driven mainly by the much higher levels of TBOEP (mean levels of TBOEP were 2.5–11,130 times higher and median levels 67–12,200 higher in suburban samples than mean and median levels of all OPEs in urban samples). If TBOEP was not included, the overall median and mean of ∑_12_OPEs and the mean levels of individual OPEs were higher in urban samples. Differences in mean levels of individual OPEs were statistically significant only for TIPP, TPP, TCEP, TDCIPP, and TEHP.

In suburban samples, non-halogenated OPEs accounted for an average of 83% of the total (73% alkyl OPEs and 10% aryl OPEs), while halogenated OPEs accounted for 17% (Table S3). In urban samples, non-halogenated OPEs made up 75% (50% alkyl OPEs and 25% aryl OPEs), while halogenated OPEs made up 25%. Differences in mean concentrations between urban and suburban samples were statistically significant (*p* < 0.05; two-sample *t*-test assuming unequal variances) only for halogenated OPEs, but not for non-halogenated OPEs.

Six of the suburban samples and nineteen of the urban samples were from homes. A comparison of concentrations in homes only showed that the median and mean of ∑_13_OPEs in urban versus suburban homes were 10,719 versus 8634 ng g^−1^ and 19,428 versus 11,790 ng g^−1^, respectively. Differences in mean concentrations were not statistically significant (*p* > 0.05; two-sample *t*-test assuming unequal variances), in agreement with the results for the comparison of overall data between urban and suburban locations. With respect to individual OPEs, differences in mean concentrations were statistically significant (*p* < 0.05; two-sample *t*-test assuming unequal variances) only for TPP and EHCPP. This probably reflects the fairly standard nature of construction and/or contents of homes in the area, regardless of the urban vs. suburban setting.

### 3.3. Comparison of Levels in Residential and Non-Residential Locations

**Figure 3:**
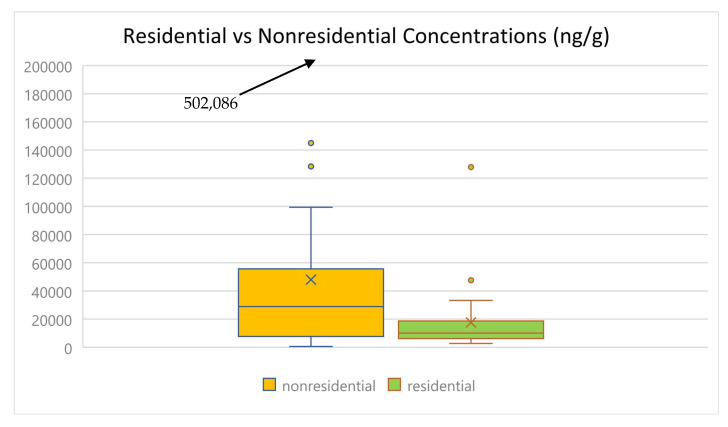
Comparison of ∑_13_OPEs concentrations in samples from residences vs. non-residences.

Thirty-nine samples were collected from non-residential locations (university offices and classrooms and daycares) and twenty-five from residences. Figure 3 shows that OPE levels were generally higher in samples from non-residential sites. The overall median and mean of ∑_13_OPEs for non-residential versus residential samples were 28,861 vs. 10,044 ng g^−1^ and 48,019 versus 17,995 ng g^−1^, respectively (Table S3). Mean levels were significantly different (*p* < 0.05; two-sample *t*-test assuming unequal variances). This is in agreement with previous studies [34,39,40,41]. The higher levels in non-residential samples may reflect the presence of more sources of OPEs in these environments (for example, more electronic equipment, more floor covering material, greater use of floor polishers). Three of four halogenated OPEs (TCEP, TCIPP, and T2CPP) were higher in residential samples, while most alkyl and aryl OPEs were present in higher concentrations in non-residential samples. These differences are reflected in the contributions of the different types of OPEs to the total (Table S3). In non-residential samples, non-halogenated OPEs accounted for 85% of the total (61% alkyl and 24% aryl OPEs), while halogenated OPEs accounted for 15%. In residential samples, non-halogenated OPEs accounted for a lower percentage of the total (62%) (43% alkyl and 19% aryl OPEs), and halogenated OPEs accounted for 38%. As noted above, chlorinated OPEs are applied to rigid and flexible polyurethane foam, so the higher levels measured in residences may reflect their emission from a variety of consumer products and furniture containing this material in residences.

### 3.4. Comparison of Levels in Apartment and Single-Family Residences

**Figure 4:**
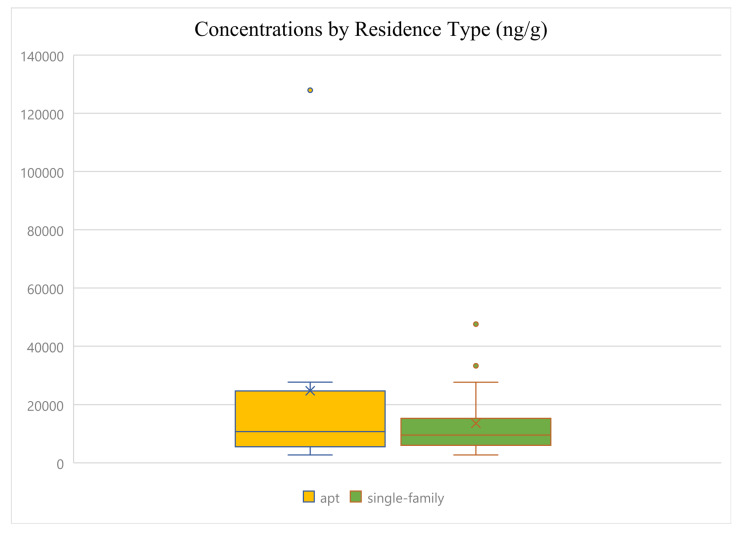
Comparison of ∑_13_OPEs concentrations by type of residence.

Cristale et al. (2018) reported that, in Brazil, median levels of OPEs were higher in samples taken in apartments than in those taken in single-family homes [27]. Sixteen dust samples in our study were collected from single-family structures (stand-alone) and nine from shared dwellings (apartments). Figure 4 shows a comparison of concentrations in both types of residence. In comparing apartments versus single-family homes, means and medians of ∑_13_OPEs were 24,778 versus 13,555 ng g^−1^ and 10,719 versus 9542 ng g^−1^, respectively. Differences in mean concentrations were not statistically significant (*p* > 0.05, two-sample *t*-test assuming unequal variances). This, along with similar medians, suggests that, in the study area, all types of residences are fairly standard in both construction materials and in contents, as expected in a modern consumer society. This is in contrast to results in Brazil, where median levels of total OPEs were considerably higher in apartments than in single-family residences [27]. Without comprehensive information on the potential number of sources of OPEs in apartments vs. single-family homes in the two study regions, it is impossible to explain the different results. Unlike the results on quantities, the profile of OPEs detected is relatively similar in this study area and the Brazil study. In both cases, TBOEP was the dominant OPE, with TDCIPP, TPHP, and TCIPP also significantly contributing to total OPEs. This suggests that regardless of quantities of sources, the types of sources are similar in both study areas.

### 3.5. Comparison of University, Daycare, and Home Sites

**Figure 5:**
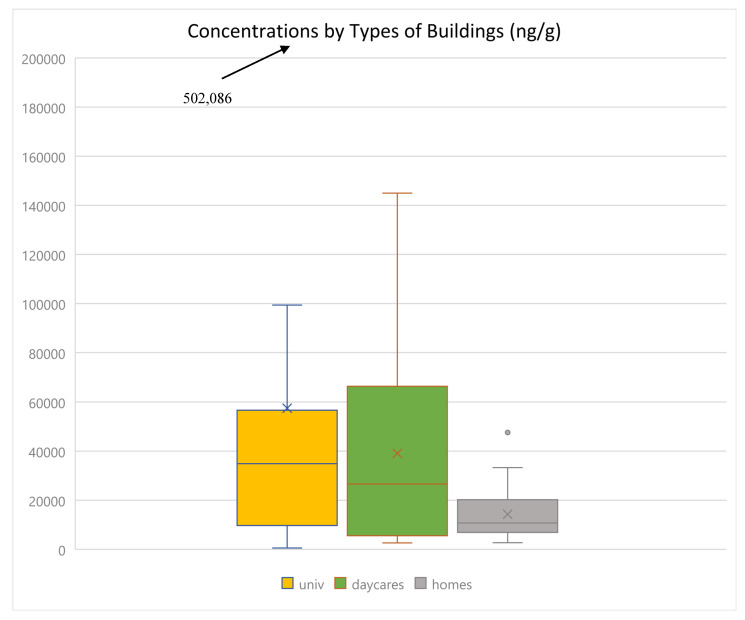
Comparison of ∑_13_OPEs concentrations between university, daycare, and residential samples.

Sampling sites included, in addition to residences, daycares (*n* = 16) and two universities (*n* = 22). Figure 5 shows a comparison of OPE mean levels between them. Mean levels of ∑_13_OPEs were in the following order: university (42,443 ng g^−1^) > daycares (39,102 ng g^−1^) > homes (17,595 ng g^−1^). Medians followed the same order. Differences in mean levels were statistically significant (*p* < 0.05, two-sample *t*-test assuming unequal variances) between homes and daycares and homes and university samples but not between university and daycare samples. These results differ from those reported for Beijing, where home levels were higher than daycares, but similar to a report from Jilkova et al. (2018), who reported the median for ∑_12_OPEs for university samples to be approximately four times higher than for residences [41,42,43,44]. Higher levels in university and daycare samples may be attributed to a higher presence of electronic devices, which are important sources of OPEs, as well as more frequent cleaning with materials containing these chemicals. Although the differences in mean levels of ∑_13_OPEs were statistically not significant between university and daycare samples, differences in mean levels of TEHP and TDCIPP were. There were differences in terms of contribution of each type of OPE to total levels (Table S3). In daycares, halogenated OPEs accounted for 6% and non-halogenated 94% (85% alkyl OPEs, 9% aryl OPEs); in university samples, halogenated OPEs made up 22% and non-halogenated OPEs 78% (44% alkyl OPEs, 34% aryl OPEs); and in residences, halogenated OPEs accounted for 36% and non-halogenated OPEs 64% (45% alkyl OPEs, 19% aryl OPEs). The greater contribution of halogenated OPEs in residences and universities may be due to the greater use of flame retardants in electronic devices such as computers, as well as laboratory and office equipment that were prevalent in these environments. The greater contribution of aryl OPEs in university samples may be due to the greater use of floor cleaning materials and lubricants in machinery, as well as more frequent application of these. However, levels of TBOEP were much higher in daycares (median of 22,940 and mean of 36,551 ng g^−1^) compared with the university (median of 4718 and mean of 27,247 ng g^−1^) and residence (median of 3710 and mean of 12,773 ng g^−1^) samples. In daycare samples, TBOEP was more dominant (contributing approximately 94% to ∑_13_OPEs) compared with samples in residences (59%) and universities (25%). The use of TBOEP as plasticizers and the presence of large quantities of plastic toys and foam/plastic items such as cribs, mats, mattresses, and pillows may explain the very high levels of this OPE in daycares [41]. TBOEP was also reported as the dominant OPE in daycares in Brazil and Sweden [27,45].

### 3.6. Influence of Age of Buildings on OPE Levels

**Figure 6:**
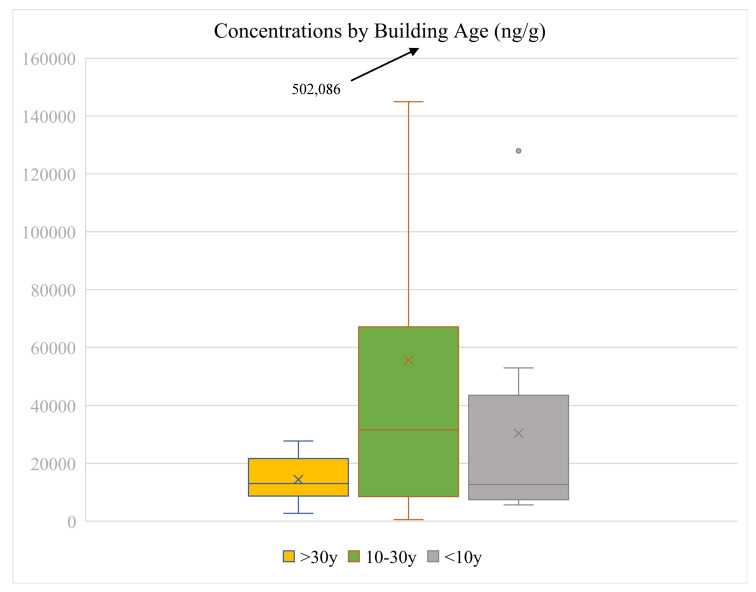
Comparison of ∑_13_OPEs concentrations in buildings of different age.

Bi et al. (2018) reported higher levels of TCIPP in dust in newer buildings compared with older ones, attributing this to the increasing use of OPEs as replacements to PBDEs; however, they also reported no correlation between other OPEs and age of buildings [4]. To test the hypothesis that newer buildings will show higher levels of OPEs to reflect the increased use of these as PBDEs were phased out, we divided buildings into age groups for those buildings where we could ascertain their year of construction. Samples were divided into those collected from “newer” (less than 10 years old, *n* = 8), “intermediate” (between 10 and 20 years old, *n* = 24), and “older” (more than 30 years old, *n* = 9) buildings, and the levels and profiles of OPEs from each were compared. Figure 6 shows the mean concentrations from samples collected at each set of buildings. Median concentrations were in the order intermediate > older~newer (Table S3). Mean concentrations were in the order intermediate > newer > older (Table S3). Mean concentrations were not significantly statistically different between the groups (*p* > 0.05, two-sample *t*-test assuming unequal variances). These results suggest that the age of the building is not the determining factor with respect to levels of OPEs in indoor environments. However, a confounding factor may be that older buildings may have been renovated more recently, so results must be interpreted with caution. Information regarding renovation was only partially available, so firm conclusions regarding this factor cannot be drawn. Levels may be more influenced by the quantities of materials/products inside that may be sources of these chemicals. In terms of types of OPEs in the three categories, the three dominant OPEs were TBOEP, TPHP, and TDCIPP, together making up a total of 85%, 92%, and 89% of the total OPEs in old, mid, and new buildings, respectively. The relative contributions of halogenated vs. non-halogenated OPEs were also similar. This suggests that the contents inside buildings may be more important than the age of the building.

### 3.7. Influence of Type of Floor on OPE Levels

**Figure 7:**
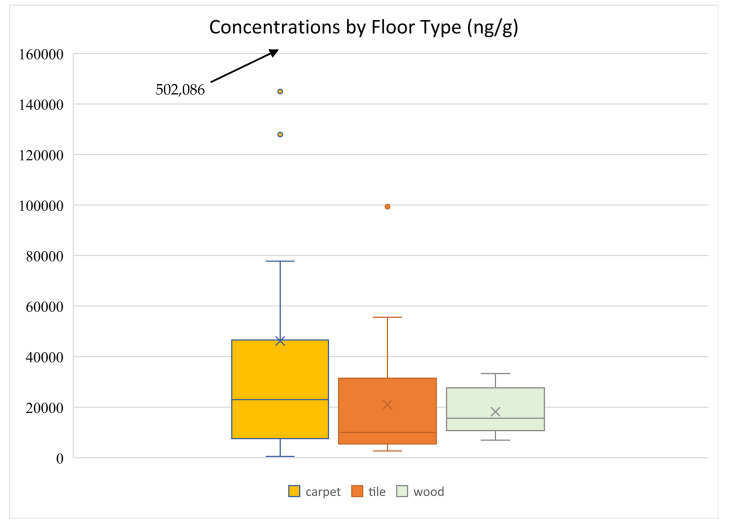
Comparison of ∑_13_OPEs concentrations by floor type.

The type of floor cover may influence levels of OPEs in indoor environments. Castorina et al. (2017) reported higher loadings of TCEP in dust in rooms with worn carpeting [38]. Bi et al. (2018) also reported that TDCIPP levels were significantly associated with the presence of carpets [4]. TDCIPP and TCIPP levels were also reported to be higher in carpeted rooms in Australia and Japan [35,46,47]. Tajima et al. (2014) reported lower levels of TBOEP and TPHP in the carpeted rooms compared with those without carpeted floors [35]. Figure 7 shows a comparison of concentrations by floor type. The mean of ∑_13_OPEs was 46,179 ng g^−1^ for samples from rooms with carpet (*n* = 38), 21,016 ng g^−1^ with tile floors (*n* = 7), and 18,223 ng g^−1^ with wood floors (*n* = 17). Means were statistically different (*p* < 0.05, two-sample *t*-test assuming unequal variances) between carpeted and wooden floors and between carpeted and tiled floors; they were not between tiled and wooden floors. Carpeted rooms had the lowest contribution of halogenated OPEs (averaging 16% of the total compared with 35% for rooms with tile floors and 29% for rooms with wood floors) and the highest contribution of alkyl OPEs (averaging 63% compared with 45% in rooms with tile and wood floors) (Table S3). These results are contrary to those cited above, which showed higher levels of halogenated OPEs in carpeted rooms. Overall, in all environments, TBOEP was the predominant OPE, with TPHP generally being the second most abundant. These results are contrary to those of [33]. Mean differences were statistically significant (*p* < 0.05, two-sample *t*-test assuming unequal variances) for TBOEP, TIPP, and TIPPP between samples from rooms with carpet and wood floors. The results suggest that the type of floor cover does influence the levels and types of OPEs in indoor environments, although the profiles in this study were different than in previous reports.

It must be stressed that comparisons between studies are likely affected by differences in sampling methodologies. For example, a factor seldom studied, if ever, is the efficiency with which vacuuming (the main method to collect dust samples from surfaces) collects dust from carpets compared with hard surfaces. Likewise, it is possible that, over time, poor efficiency with respect to cleaning dust from carpets results in a greater accumulation of contaminated dust than one might find for hard surfaces, which may be cleaned more efficiently.

### 3.8. Comparison of Home Levels to Other Locations

Concentrations in homes in this study were compared with values reported for homes in other locations around the world (Table 2, with references), with the caveat that comparisons may be difficult due to differences in sampling methodology. In this study, the range of ∑_13_OPEs in homes was from 545 ng g^−1^ to 502,086 ng g^−1^, with a median of 15,447 ng g^−1^. Overall, the concentrations measured in this study were comparable to those reported for other developing countries and generally higher than in developing countries. Results suggest that the quantities of OPEs used in products in the Tampa Bay (Florida) area are similar to other developed regions of the world.

### 3.9. Exposure Calculations

Exposure rates were calculated using the equation below, as in Shoeib et al. (2019) [7].

*EDI* = *C* × *I_dust_*

1000 × *bw*

where *EDI* = expected daily intake of target OPEs per kg of body weight (ng kg^−1^ *bw* day^−1^), *C* = concentration of OPEs in dust (ng g^−1^), *I_dust_* = dust ingestion rate (mg day^−1^), and *bw* = body weight (kg). Calculations were carried out using median OPE concentrations coupled with mean dust ingestion rates to represent typical exposure levels of the majority of the population, and the upper 95th percentile of OPE concentrations coupled with high dust ingestion rates to represent high exposure rates that may apply to a small portion of the population. The following values were used: average body weight of 12.3 kg for toddlers, mean and high dust ingestion rates of 20 and 50 mg day^−1^, respectively; and for adults an average body weight of 70 kg and mean and high dust ingestion rates of 50 and 200 mg day^−1^ [2,27,49]. One hundred percent of dust intake absorption was assumed. Values for toddlers were calculated only for residences and daycares, since it is not likely that toddlers spend any time in university rooms.

The sum EDI values (ng kg^−1^ bw day^−1^) were 12 and 552 in toddlers and 6 and 451 in adults in the average and high exposure scenarios (Table S4). Thus, values were approximately 1.5–2 times higher in toddlers. Table S4 shows that the sum EDI value in the mean exposure scenario for toddlers was comparable to values reported for Washington (USA), Vancouver, and Istanbul and lower than those reported for Beijing, Germany, Sweden, Brazil, and Cairo; for adults it was higher than values reported for Washington and Istanbul, comparable to values for Beijing, Sweden, Germany, and Cairo, and lower than values for Brazil and Vancouver [7,16,27,57,58,59]. In the high exposure scenario, for toddlers, the sum of EDI was higher than values reported for Washington, Beijing, and Istanbul and lower than values for Germany, Sweden, Brazil, Vancouver, Cairo, the U.K., and Norway; for adults, the value was comparable to Brazil and Vancouver and higher than values for Washington, Beijing, Germany, Sweden, Cairo, and Istanbul [2,7,16,27,57,58,59]. The highest intake contributions were for TBOEP, accounting for 60% and 83% of the total in toddlers and 59% and 72% in adults in the mean and high exposure scenarios, respectively. TPHP, TDCIPP, and TCIPP were the next most significant contributors to intake in both toddlers and adults. Table 3 includes available reference dose (RfD) values [7,54]. Only in the case of TBOEP in the high exposure scenario was the relative percent intake of the RfD significant: 31% for toddlers and 22% for adults.

## 4. Conclusions

This work presents the first investigation of the levels of OPEs in indoor dust in different environments in the Tampa Bay area of Florida. The findings showed that the ranges of concentrations of 13 OPEs measured in this study at the time of study were generally in line with those reported for other locations in the U.S., Canada, Europe, and Japan, although median values were generally lower. This suggests that there was a transition in the use of OPEs as replacements for PBDEs in the study area. Overall, non-halogenated OPEs were predominant, with TBOEP contributing on average to 51% of total OPEs. Other OPEs measured in higher concentrations were TPHP and the chlorinated OPEs TDCIPP, T2CPP, and TCIPP. Median levels were significantly higher in suburban samples compared with urban samples, although this was driven primarily by the high levels of TBOEP in suburban samples; when this OPE was excluded, levels were significantly higher in urban samples. There were measurable differences when comparing different environments (e.g., residences versus non-residences, daycares and universities versus residences, rooms with carpets versus rooms with other floor coverings). Estimated daily intakes of OPEs in the study area were generally in line with or lower than values reported for other locations, and well below reported RfD values known for certain compounds. The results must be taken in the context that different studies have used different methodologies in sampling, processing, and analysis. As noted previously, for example, studies looking at the efficiency of dust collection by vacuums from different surfaces may be important to standardize results. It highlights the need for the standardization of methodologies in this area, and in environmental work in general. These results are important data from a region that has not been studied before. As noted above, it is important to have comprehensive data from all regions of the world to have a truly global understanding of the cycling and potential effect of these chemicals. As concern about OPEs has increased due to their association with various health effects, there have been increasing calls for phasing them out. This study can thus serve as important baseline information for the study region for future studies measuring the effect of any such decrease in use.

## Figures and Tables

**Table 1:** Overall OPE concentrations (ng g^−1^) and detection frequency (%).

OPE	Range (min–max)	Mean	Median	DF ^a^ (%)
TIPP	BD ^b^—18	3.48	2	86
TPP	BD—26	5.95	5	84
TNBP	BD—4.789	223	41	92
TCEP	5—2199	181	85	100
TCIPP	18—18,807	937	360	100
T2CPP	17—27,212	1211	458	100
TDCIPP	BD—23,898	1775	530	98
TPHP	BD—210,108	7614	1046	98
TBOEP	BD—250,904	22,672	4641	88
EHDPP	15—1484	178	109	100
TEHP	BD—1741	239	76	98
TIPPP	BD—621	42	38	36
TMPP ^c^	BD—34,131	1052	54	50 ^d^
∑_13_OPEs	545–502,086	36,135	15,447	

^a^ DF—detection frequency; ^b^ BDL—below detection limit; ^c^ value is sum of the o, m, and p isomers of tricresyl phosphate; ^d^ detection means at least one of the three isomers was measured.

**Table 2:** Comparison of ∑OPE concentrations (ng g^−1^) in homes.

Location	Year of Sampling	n	No of OPEs in ∑OPEs	Median	Range	Ref.
Tampa Bay, USA	2016	25	13	15,447	545–502,086	This study
Albany, USA	2018	8	15	30,600	16,200–224,000	A
Texas, USA	2014	92	5	19,300	8240–1,220,000	B
Egypt	2012–2013	20	8	189	38–962	C
New Zealand	2010	34	7	5510		D
Kuwait	2011	15	12	6550	2260–146,900	E
Pakistan	2011	15	12	575	65–900	E
Netherlands	2012	8	9	27,000	7400–167,000	F
Germany	2010–2011	6	7		800–6000	G
Spain		5	10	10,121	5223–20,851	H
Romania	2010	47	9	7890		I
China (urban)	2013–2014	11	12	9340	4450–27,500	J
China (rural)	2013–2014	25	12	7480	2260–20,700	J
China		20	14	9200	2060–19,950	K
Belgium	2008	33	9	13,100	1920–94,700	L
Canada	2008	92	11	41,400	2600–733,300	M
Turkey	2012	39	11	2400	1100–61,900	M
Egypt	2013–2014	17	11	14,000	7300–99,500	M
U.K.	2013	10	10	79,000		N
Norway	2013–2014	10	10	23,000		N
Nepal	2014	28	8	732	153–12,100	O
Saudi Arabia		15	8	3750	1000–13,800	P
Japan	2009–2010	10	9	97,000	9300–1,100,000	Q
Norway		61	9	20,500	3662–505,000	R

A = [6], B = [4], C = [42], D = [48], E = [49], F = [50], G = [36], H = [27], I = [51], J = [52], K = [53], L = [54], M = [7], N = [2], O = [55], P = [56], Q = [33], R = [32].

**Table 3:** Daily intake of toddlers and adults at mean and high exposure rate scenarios (ng kg^−1^ bw day^−1^).

OPE		Toddler ^a^		Adult	
	RfD	Mean	High	Mean	High
TIPP		0.003	0.044	0.001	0.031
TPP		0.009	0.071	0.003	0.050
TNBP		0.068	4.33	0.029	2.98
TCEP	2200	0.117	1.67	0.061	1.24
TCIPP	8000	0.681	14.7	0.257	9.72
T2CPP		0.801	18.5	0.327	12.3
TDCIPP	1500	0.526	16.4	0.379	12.7
TPHP	7000	0.991	28.6	0.747	71.3
TBOEP	1500	7.42	462	3.32	326
EHDPP		0.155	1.21	0.078	1.01
TEHP		0.081	1.19	0.054	4.01
TIPPP		0.139	1.19	0.027	0.724
ToCP		1.15	1.63	0.246	2.13
TmCP		0.047	0.133	0.031	0.775
TpCP		0.030	0.133	0.026	5.17
TOTAL		12.2	552	5.58	451

^a^ Calculations based on residence and daycare samples (excluding university samples).

## Data Availability

Data are contained within the article or Supplementary Materials.

## Supplementary Materials

The following supporting information can be downloaded at: https://www.mdpi.com/article/10.3390/toxics13060508/s1, Table S1. Characteristics of sampling sites; Table S2. OPE levels in Tampa Bay samples; Table S3. Comparison of OPE levels in different environments (ng g^−1^); Table S4. Comparison of exposure values (ng kg^−1^ bw day^−1^).
