# Supplementary material for: Organophosphate Flame Retardants in Indoor Dust in the Tampa Bay (Florida) Area
Source: Toxics. 2025 Jun 16;13(6):508. doi: 10.3390/toxics13060508 (PMC12197667; doi:10.3390/toxics13060508)
Supplement: Supplementary file 1 [file toxics-13-00508-s001.zip › toxics-3623364-supplementary.pdf]

Supporting Information

Organophosphate Flame Retardants in Indoor Dust in the Tampa Bay (Florida) Area

Adebayo Solanke [1,2], Lukasz Talalaj [3], Claire Graham [1] and Henry Alegria [1,*]

[1] Department of Environmental Science, Geography & Policy, University of South Florida St. Petersburg, 140 7th Avenue South, St. Petersburg, FL 33701, USA; adebayo.solanke@fdacs.gov (A.S.); clairegraham514@gmail.com (C.G.)
[2] School of the Environment, Florida A&M University, 1601 Martin L. King Jr. Blvd., Tallahassee, FL 32307, USA
[3] Environmental Protection Commission of Hillsborough County, 3629 Queen Palm Drive, Tampa, FL 33619, USA; ltalalaj@usf.edu
* Correspondence: halegria@usf.edu

Table S1. Characteristics of sampling sites.

**Sample**					**Floor cover**	**age**
Sample 1	Urban	residential	single-family		tile floor	intermediate
Sample 2	Urban	nonresidential			carpet	
Sample 3	Urban	residential	single-family		tile floor	old
Sample 4	Urban	residential	single-family		tile floor	
Sample 5	Urban	residential	single-family		wood floor	old
Sample 6	Urban	nonresidential			wood floor	
Sample 7	Suburban	residential	single-family		carpet	newer
Sample 8	Urban	residential	single-family		wood floor	old
Sample 9	Urban	residential	single-family		wood floor	intermediate
Sample 10	Suburban	residential	single-family		wood floor	newer
Sample 11	Urban	residential	apartment		carpet	intermediate
Sample 12	Urban	residential	single-family		carpet	old
Sample 13	Urban	residential	apartment			old
Sample 14	Urban	residential	apartment		carpet	intermediate
Sample 15	Suburban	residential	single-family		wood floor	intermediate
Sample 16	Urban	residential	single-family		tile floor	old
Sample 17	Suburban	residential	apartment		carpet	intermediate
Sample 18	Urban	residential	apartment		carpet	old
Sample 19	Urban	nonresidential		university	carpet	intermediate
Sample 20	Urban	nonresidential		university	carpet	intermediate
Sample 21	Urban	nonresidential		university	carpet	intermediate

Sample 22	Urban	nonresidential		university	carpet	intermediate
Sample 23	Urban	nonresidential		university	carpet	intermediate
Sample 24	Urban	nonresidential		university	carpet	intermediate
Sample 25	Urban	residential	single-family		carpet	old
Sample 26	Urban	residential	apartment		wood floor	old
Sample 27	Urban	nonresidential		university		intermediate
Sample 28	Urban	nonresidential		university	carpet	intermediate
Sample 29	Urban	nonresidential		university	carpet	intermediate
Sample 30	Urban	nonresidential		university	carpet	intermediate
Sample 31	Urban	nonresidential		university	carpet	intermediate
Sample 32	Urban	nonresidential		university	carpet	intermediate
Sample 33	Urban	nonresidential		university	carpet	intermediate
Sample 34	Urban	residential	apartment		tile floor	
Sample 35	Urban	residential	single-family		tile floor	
Sample 36	Urban	residential	single-family		tile floor	
Sample 37	Urban	residential	single-family		tile floor	
Sample 38	Urban	nonresidential		university	tile floor	newer
Sample 39	Urban	nonresidential		university	tile floor	newer
Sample 40	Urban	nonresidential		university	tile floor	newer
Sample 41	Urban	nonresidential		university	carpet	
Sample 42	Urban	nonresidential		university	tile floor	newer
Sample 43	Urban	nonresidential		university	tile floor	newer
Sample 44	Urban	nonresidential		university	tile floor	
Sample 45	Urban	nonresidential		university	tile floor	
Sample 46	Urban	residential	apartment		carpet	newer
Sample 47	Suburban	residential	apartment		tile floor	
Sample 48	Suburban	residential	single-family		tile floor	
Sample 49	Suburban	nonresidential		daycare	carpet	intermediate
Sample 50	Suburban	nonresidential		daycare	carpet	intermediate
Sample 51	Suburban	nonresidential		daycare	carpet	intermediate
Sample 52	Suburban	nonresidential		daycare	carpet	
Sample 53	Suburban	nonresidential		daycare	carpet	intermediate
Sample 54	Urban	nonresidential		daycare	carpet	intermediate
Sample 55	Urban	nonresidential		daycare	carpet	
Sample 56	Urban	nonresidential		daycare	carpet	
Sample 57	Suburban	nonresidential		daycare	carpet	
Sample 59	Urban	nonresidential		daycare	carpet	
Sample 60	Urban	nonresidential		daycare	carpet	
Sample 61	Urban	nonresidential		daycare	carpet	
Sample 62	Urban	nonresidential		daycare	carpet	
Sample 63	Urban	nonresidential		daycare	carpet	

Sample 64	Urban	nonresidential		daycare	carpet	
Sample 65	Urban	nonresidential		daycare	carpet	

Note: Sample 58 was accidentally destroyed.

Table S2. OPEs levels in Tampa Bay samples.

	Alkyl	Alkyl	Alkyl	Halog	Halog	Halog	Halog	Aryl	Alkyl	Aryl	Alkyl	Aryl	Aryl	Aryl	Aryl	Aryl	
**Sample**	**TiPP**	**TPrP**	**TNBP**	**TCEP**	**TCIPP**	**TCPP**	**TDCPP**	**TPHP**	**TBOEP**	**EHDPP**	**TEHP**	**ToCP**	**TmCP**	**TpCP**	**T2PPP**	**TMPP**	**ΣOPE**
Sample 1	10.9	0.1	82.5	15.7	182.1	172.7	178.6	165.9	1988.8	46.7	61.3	BD	23.5	BD	BD	23.5	2929
Sample 2	7.8	0.9	18.6	69.4	502.5	515.1	16104.2	671.1	1954.9	111.3	43.9	BD	BD	BD	122.0	BD	20122
Sample 3	15.9	7.9	146.6	82.3	714.2	726.4	181.6	4029.2	3534.7	301.1	BD	BD	BD	BD	304.0	BD	10044
Sample 4	15.1	2.4	63.4	29.2	100.7	113.0	268.4	291.0	1205.7	235.7	375.8	BD	BD	BD	BD	BD	2701
Sample 5	4.7	0.8	20.1	117.9	314.4	385.8	7864.4	16135.2	2119.3	165.2	223.0	BD	272.9	24.9	40.4	297.9	27689
Sample 6	5.1	2.0	1173.0	219.9	3621.2	4576.6	62.5	BD	BD	142.9	78.5	10360.6	BD	BD	BD	10360.6	20242
Sample 7	4.9	1.8	53.8	339.2	1089.4	1305.7	728.9	830.8	4565.1	59.4	43.0	BD	BD	17.6	BD	17.6	9040
Sample 8	10.1	3.9	10.7	1240.5	289.6	338.9	3123.9	1898.9	8358.9	100.8	264.0	BD	BD	BD	BD	BD	15640
Sample 9	1.3	8.9	16.1	51.0	886.2	1090.6	459.5	716.5	3349.1	77.1	293.7	BD	BD	11.4	BD	11.4	6961
Sample 10	7.0	4.7	240.1	49.8	503.6	618.3	408.6	2249.4	5527.2	71.8	96.1	973.7	BD	BD	BD	973.7	10750
Sample 11	8.5	10.3	27.3	99.0	435.5	446.2	880.1	1851.7	2212.9	81.2	134.5	BD	BD	37.0	621.2	37.0	6845
Sample 12	3.9	12.2	0.0	54.7	319.2	386.6	323.3	1518.6	4523.8	106.5	73.8	BD	BD	BD	BD	BD	7322
Sample 13	0.4	0.3	23.2	102.9	465.9	524.2	854.8	486.7	BD	159.3	99.3	BD	BD	BD	BD	BD	2717
Sample 14	8.8	21.0	23.7	407.5	4163.2	5558.8	BD	1188.2	9783.6	355.0	102.4	BD	33.8	BD	4.2	33.8	21650
Sample 15	3.0	10.0	246.2	55.7	389.4	492.5	389.5	4221.3	27325.8	94.2	34.1	BD	BD	8.2	BD	8.2	33270
Sample 16	0.0	7.5	19.7	75.0	758.6	909.6	4016.2	205.2	5047.8	63.8	147.3	BD	BD	BD	BD	BD	11251
Sample 17	0.0	0.4	91.0	160.3	1913.7	2342.2	513.3	2152.6	979.2	64.1	11.4	BD	BD	BD	BD	BD	8228
Sample 18	0.6	7.2	1108.9	32.3	359.4	469.8	4040.8	7653.7	13551.1	99.9	245.9	BD	BD	161.2	BD	161.2	27731
Sample 19	7.7	8.0	14.3	55.9	482.6	566.6	578.9	14738.7	25583.5	164.8	23.3	BD	BD	1313.5	BD	1313.5	43538
Sample 20	3.5	0.0	58.7	78.9	612.6	704.6	988.2	45724.5	BD	224.2	281.8	26.1	54.6	8894.0	BD	8974.7	57652
Sample 21	1.4	0.0	34.8	54.2	751.9	793.9	4500.8	14963.7	54330.3	300.2	92.7	0.6	20.2	1899.0	37.7	1919.7	77781
Sample 22	0.2	12.5	11.3	26.0	196.7	227.9	341.7	1702.7	32145.6	104.4	31.9	BD	BD	60.1	BD	60.1	34861
Sample 23	1.8	0.0	1772.9	89.2	490.9	505.1	3205.8	26512.2	BD	138.5	57.9	15.5	362.5	319.1	22.2	697.1	33494
Sample 24	1.6	7.4	111.5	255.1	966.6	1095.8	4232.3	210107.7	250903.7	175.0	73.3	246.2	262.8	33622.0	24.9	34130.9	502086
Sample 25	1.7	25.8	40.5	31.1	1072.1	1302.2	296.0	3351.8	7843.9	76.7	110.6	BD	BD	BD	BD	BD	14152
Sample 26	7.3	10.8	10.0	439.0	999.6	1242.2	3192.0	304.4	6640.3	83.1	43.8	BD	BD	BD	36.0	BD	13008

Sample 27	8.2	21.5	29.1	163.1	241.4	296.6	1498.6	55692.8	8536.6	115.9	92.3	BD	4.0	3589.9	BD	3593.9	70290
Sample 28	0.5	0.7	5.2	5.4	18.0	16.6	78.7	122.0	218.5	14.5	64.4	BD	BD	BD	BD	BD	545
Sample 29	2.1	2.3	57.3	88.6	325.8	487.7	2336.1	5790.7	4717.6	1483.8	1639.9	BD	BD	BD	22.1	BD	16954
Sample 30	1.7	12.8	46.3	382.3	211.3	241.9	1108.4	4848.5	3012.5	80.9	122.2	BD	48.7	BD	0.1	48.7	10118
Sample 31	0.7	0.8	31.3	91.0	301.8	93.9	576.5	336.6	919.8	71.0	1530.0	BD	37.4	BD	BD	37.4	3991
Sample 32	7.9	0.6	19.3	76.9	198.3	245.3	1455.0	1437.7	3914.7	168.7	1741.4	BD	BD	BD	BD	BD	9266
Sample 33	2.6	0.0	50.6	139.3	319.0	411.4	23897.7	9989.0	3092.4	68.0	1433.5	17.7	492.2	BD	263.9	509.9	40177
Sample 34	0.0	10.7	32.9	101.9	2854.6	3619.5	242.2	505.0	2599.6	95.4	623.4	BD	BD	33.6	BD	33.6	10719
Sample 35	6.0	17.8	60.7	241.3	18807.3	27216.2	446.6	609.6	BD	94.3	36.1	BD	BD	15.2	16.5	15.2	47568
Sample 36	4.6	2.0	6.4	119.4	572.5	718.3	273.0	1746.8	2896.3	50.5	28.6	BD	BD	2.0	BD	2.0	6420
Sample 37	0.0	10.1	69.5	411.2	1110.3	1387.4	784.2	512.4	1374.9	176.6	BD	27.3	BD	BD	BD	27.3	5864
Sample 38	17.6	5.7	29.2	41.5	111.5	143.1	554.6	291.2	12500.1	97.2	761.8	BD	BD	BD	56.6	BD	14610
Sample 39	3.6	15.8	53.2	2199.4	249.7	251.3	463.9	1122.4	47664.1	274.9	688.3	BD	BD	BD	BD	BD	52986
Sample 40	0.3	6.0	12.4	256.0	239.5	310.0	2958.3	1642.0	BD	122.5	82.1	BD	BD	BD	BD	BD	5629
Sample 41	BD	3.8	0.0	362.6	267.8	319.5	3660.9	3366.2	47108.4	343.3	202.6	BD	27.4	BD	19.8	27.4	55682
Sample 42	0.1	BD	464.1	56.7	346.5	388.3	2569.1	7944.7	BD	1348.0	450.7	1647.7	BD	38.3	BD	1686.0	15254
Sample 43	0.9	0.6	103.7	110.4	229.8	285.1	1946.8	3368.9	BD	240.4	34.8	572.8	BD	BD	BD	572.8	6894
Sample 44	2.7	BD	25.0	282.6	2215.3	2740.3	4080.0	5897.8	38821.4	239.7	343.4	775.6	137.9	7.6	BD	921.2	55569
Sample 45	1.2	4.7	133.1	879.2	318.2	397.8	913.0	1687.8	93568.4	237.3	1237.1	BD	BD	BD	BD	BD	99378
Sample 46	BD	10.4	68.8	140.4	3497.4	4405.5	127.6	1055.2	117882.0	223.7	32.5	BD	BD	BD	478.8	BD	127922
Sample 47	BD	1.7	59.6	71.7	404.5	480.7	785.3	460.7	1854.3	34.2	28.6	BD	BD	BD	BD	BD	4181
Sample 48	2.5	4.6	26.6	111.9	822.7	1042.8	110.3	760.1	2061.1	37.0	281.9	BD	BD	BD	11.0	BD	5272
Sample 49	BD	3.8	0.0	30.4	226.8	291.1	155.0	358.7	28741.6	89.8	51.0	BD	BD	BD	19.9	BD	29968
Sample 50	1.6	6.7	10.7	25.9	74.1	99.9	197.1	513.7	143502.0	382.7	151.4	BD	BD	BD	BD	BD	144966
Sample 51	BD	5.4	0.0	43.0	239.3	322.4	258.3	367.2	75713.1	138.5	66.9	BD	BD	BD	BD	BD	77154
Sample 52	1.1	BD	19.2	123.2	418.8	502.2	1003.6	1036.9	124717.8	271.5	77.8	BD	BD	19.3	115.3	19.3	128307
Sample 53	2.2	BD	281.5	8.6	50.5	72.9	177.2	251.6	23074.7	201.1	39.5	BD	BD	BD	194.0	BD	24354
Sample 54	1.5	BD	14.4	37.6	58.4	87.6	212.3	424.3	6191.7	179.9	26.9	BD	BD	BD	BD	BD	7235
Sample 55	4.5	11.5	4788.6	139.1	414.5	486.1	289.7	301.2	22806.1	60.1	49.0	BD	BD	BD	BD	BD	29350
Sample 56	1.4	BD	552.3	23.7	97.8	117.6	89.5	118.4	32779.1	78.7	27.0	BD	BD	BD	173.2	BD	34059

Sample 57	1.0	BD	383.9	26.1	360.3	440.3	324.9	699.2	74830.1	226.6	136.4	BD	3.9	BD	BD	3.9	77433
Sample 59	2.2	5.1	666.9	68.0	306.5	357.5	547.2	478.3	5079.9	111.6	33.1	BD	BD	BD	BD	BD	7656
Sample 60	1.8	5.6	5.6	8.6	57.0	76.5	83.4	450.8	3192.3	30.1	11.3	BD	BD	BD	BD	BD	3923
Sample 61	BD	9.4	BD	153.8	467.4	593.3	695.1	7336.3	9567.6	94.2	31.5	442.9	354.8	BD	6.8	797.6	19753
Sample 62	2.6	11.4	284.5	203.2	488.5	586.0	355.1	324.6	26496.3	58.6	50.0	BD	BD	BD	BD	BD	28861
Sample 63	2.0	3.1	42.0	5.5	43.2	52.9	103.5	834.7	1483.3	29.4	7.9	BD	5.4	BD	BD	5.4	2613
Sample 64	2.0	8.5	375.3	60.4	196.4	241.3	286.5	401.4	3311.9	99.6	19.7	BD	BD	BD	BD	BD	5003
Sample 65	2.8	10.7	74.7	50.6	230.3	269.3	194.3	564.7	3331.0	145.3	40.0	BD	BD	BD	85.3	BD	4999
**mean**	**3.5**	**5.9**	**223.5**	**180.8**	**937.1**	**1211.0**	**1774.6**	**7614.4**	**22672.4**	**178.4**	**239.4**	**236.0**	**33.5**	**782.4**	**41.8**	**1051.9**	**36135**
**stdev**	**4.0**	**5.9**	**651.3**	**322.8**	**2406.4**	**3454.2**	**3670.4**	**27246.3**	**42632.3**	**238.6**	**406.6**	**1302.8**	**96.8**	**4309.7**	**110.3**	**4518.7**	**66964**
**range**	**0 - 18**	**0 - 26**	**0 - 4789**	**5.4 - 2199**	**18 - 18807**	**17 - 27216**	**0 - 23898**	**0 - 210108**	**0 - 250904**	**15 - 1484**	**0 - 1741**	**0 - 10361**	**0 - 492**	**0 - 33622**	**0 - 621**	**0 - 34131**	**525 - 502086**
**median**	**2.0**	**4.7**	**41.3**	**85.4**	**359.9**	**458.0**	**530.2**	**1046.1**	**4641.4**	**108.9**	**75.8**	**0.0**	**0.0**	**0.0**	**0.0**	**54.4**	**15447**

Table S3. Comparison of OPE levels in different environments (ng g^{-1}).

Environment	Mean (+/- stdev)	Median	Range	Contribution by Type
Overall	36,135 (+/- 66,964)	15,447	545 – 502,086	23% halog, 77% nonhalog (55% alkyl, 22% aryl)
Suburban	46,077 (+/- 47,309)	27,161	4,181 – 144,966	17% halog, 83% nonhalog (73% alkyl, 10% aryl)
Urban	33,840 (+/- 69,861)	14,932	545 – 502,086	25% halog, 75% nonhalog (50% alkyl, 25% aryl)
Nonresidential	48,019 (+/- 81,236)	28,861	545 - 502,086	15% halog, 85% nonhalog (61% alkyl, 24% aryl)
All Residences	17,595 (+/- 24,910)	10,044	2,701 – 127,922	36% halog, 64% nonhalog (45% alkyl, 19% aryl)
Apartments	24,778 (+/- 37,267)	10,719	2,717 – 127,922	42% halog, 58% nonhalog (42% alkyl, 16% aryl)
Single-family	13,555 (+/- 11,957)	9,542	2,701 – 47,568	33% halog, 67% nonhalog (47% alkyl, 20% aryl)
Universities	54,223 (+/- 98,982)	34,861	545 - 502,086	22% halog, 78% nonhalog (44% alkyl, 34% aryl)
Daycares	39,102 (+/- 43,216)	26,607	2,613 – 144,966	6% halog, 94% nonhalog (85% alkyl, 9% aryl)
All Residences	17,595 (+/- 24,910)	10,044	2,701 – 127,922	36% halog, 64% nonhalog (45% alkyl, 19% aryl)
Bldg age: >30y	14,395 (+/- 7,981)	13,008	2,717 – 27,731	33% halog, 67% nonhalog (42% alkyl, 25% aryl)
Bldg age:10-20 y	55,605 (+/- 98,898)	31,619	545 – 502,086	17% halog, 83% nonhalog (56% alkyl, 27% aryl)
Bldg age: <10 y	30,36 (+/- 39,541)	12,680	5,629 – 127,192	25% halog, 75% nonhalog (49% alkyl, 16% aryl)
Carpet floors	46,179 (+/- 83,141)	23,002	545 – 502,086	16% halog, 84% nonhalog (63% alkyl, 21% aryl)
Tile floors	21,016 (+/- 26,043)	15,640	2,701 – 99,378	35% halog, 65% nonhalog (45% alkyl, 20% aryl)
Wood floors	18,2230(+/- 8,756)	10,044	6,961 – 33,270	29% halog, 71% nonhalog (45% alkyl, 26% aryl)

Table S4. Comparison of exposure values (ng kg^{-1} bw day^{-1}).

Reference	Toddlers		Adults	
	mean	high	mean	high
Tampa Bay	12.2	552	5.58	451
Washington, USA [55]	18	34.3	1.6	9.9
Beijing [16]	35	91	6.7	33
Germany [57]	60	1201	7.8	150
Sweden [56]	200	4450	9.7	135
Brazil [25]	950	3800	21	520
Vancouver [7]	15	2939	19.4	523

Cairo [7]	36	845	6.4	150
Istanbul [7]	9.4	240	1.6	43
U.K. [2]		17,900		
Norway [2]		1,600		
